# Readmission Following Respiratory Syncytial Virus Hospitalization among Children <5 Years of Age

**DOI:** 10.1093/jpids/piaf036

**Published:** 2025-04-29

**Authors:** Yoonyoung Choi, Evan Heller, Linda Amoafo, Yue Zhang, Kaleb Miller, Abbey Loveridge, Madelyn Ruggieri, Per Gesteland, Krow Ampofo

**Affiliations:** Value & Implementation, Merck & Co., Inc., Rahway, NJ, United States; Department of Pediatrics, University of Utah, Salt Lake City, UT, United States; Department of Population Health Sciences, University of Utah, Salt Lake City, UT, United States; Department of Population Health Sciences, University of Utah, Salt Lake City, UT, United States; Department of Pediatrics, University of Utah, Salt Lake City, UT, United States; Department of Pediatrics, University of Utah, Salt Lake City, UT, United States; Value & Implementation, Merck & Co., Inc., Rahway, NJ, United States; Department of Pediatrics, University of Utah, Salt Lake City, UT, United States; Department of Pediatrics, University of Utah, Salt Lake City, UT, United States

**Keywords:** readmission, Respiratory Syncytial Virus, influenza virus, human metapneumovirus, lower respiratory infection hospitalization

## Abstract

**Background:**

Hospitalization with lower respiratory infection (LRI) by Respiratory Syncytial Virus (RSV) and other respiratory viruses is common in young children. However, the likelihood of readmission following RSV LRI compared to other common respiratory viral infections is unknown.

**Methods:**

This prospective study included children <5 years and hospitalized with laboratory-confirmed RSV LRI at two hospitals in Salt Lake City, Utah, from October 31, 2019 to April 30, 2022. For comparison, we retrospectively identified children <5 years, hospitalized during the same period with Influenza virus (IV) or human metapneumovirus (hMPV) LRI. Readmissions were tracked for 1.5 years post-discharge. We calculated the incidence proportion of readmissions and estimated hazard ratios using Cox proportional hazards model with Covariate Balancing Propensity Score.

**Results:**

Among children hospitalized with RSV, IV, and hMPV LRI, all-cause hospital readmission was common, with 30-day readmission proportions ranging between 5% and 9% and increasing to between 19% and 30%, 1.5 years post-discharge. Respiratory-related readmission varied by virus, with RSV having higher proportions, increasing to 16.8% 1.5 years post-discharge, compared to 6%-7% with IV and hMPV. After adjusting for confounders, RSV hospitalization was associated with an increased hazard of respiratory-related readmission within 1.5 years after hospitalization compared to IV (HR 3.62, 95% CI, 1.13-11.64) or hMPV (HR 3.56, 95% CI, 1.14-11.06).

**Conclusion:**

Respiratory-related readmission proportion was higher and progressive over time among children <5 years with an index RSV admission compared to IV and hMPV. This underscores the critical need for prevention of RSV infection in infants and young children through RSV immunization strategies.

## Teaser

Children < 5 years hospitalized with RSV face a fourfold higher risk of being readmitted for respiratory issues within 1.5 years compared to those hospitalized with influenza or human metapneumovirus. This emphasizes the need for widespread RSV immunoprophylaxis.

## INTRODUCTION

Respiratory Syncytial Virus (RSV) is one of the most common causes of respiratory infection worldwide.^1-3^ By the first year of life, about 70% of children experience at least one RSV infection, and depending upon the severity of subsequent seasonal RSV epidemics, 30%-75% of children will have a second RSV infection by 2 years of age.^4-6^ Every year in the United States, it is estimated that over 2 million children <5 years of age seek medical care for RSV infection, resulting in 1.5 million outpatient visits, 500 000 emergency department visits and 50 000 hospitalizations,^5,7,8^ the majority among children <2 years of age.^5^

There are short- and long-term consequences associated with RSV infection in children. A number of population-based studies have reported a temporal association between community RSV activity and hospital admissions with invasive pneumococcal disease (IPD) in children, with the association stronger than that of influenza virus (IV) and human metapneumovirus (hMPV) community activity.^9-12^ Long-term consequences of recurrent wheezing and asthma have been reported after RSV and rhinovirus (RV) LRI.^13-16^ The majority (~90%) of pediatric asthma diagnosis are made before 6 years of age, with 70% of children reporting reactive airway disease-like symptoms after a history of RSV and RV hospitalization.^16-18^

Healthcare resource utilization is common during and after confirmed lower respiratory infection (LRI)’s in young children,^19^ however, the risk and characterization of readmission after LRI hospitalization by respiratory viral type has not been studied. We conducted a prospective cohort study of children <5 years-of-age, who were hospitalized with laboratory-confirmed LRI. The aim was to assess the association between hospitalization due to RSV LRI and subsequent readmission, in comparison to children with IV and hMPV LRI hospitalization.

## METHODS

### Study Design, Setting, and Participants

We prospectively identified children aged 1 week to <5 years, who resided in Salt Lake County, Utah, and were hospitalized with laboratory-confirmed RSV LRI at Primary Children’s and Riverton hospitals in Salt Lake City, Utah from October 31, 2019 to April 30, 2022 (hereafter referred to as the RSV-index season). At the study hospitals, respiratory viral testing was required as part of standard-of-care for all children hospitalized with respiratory symptoms for the purposes of bed cohorting and appropriate isolation. Additionally, respiratory viral testing was recommended for all infants younger than 90 days undergoing evaluation for fever.

Respiratory viral testing was performed using nasopharyngeal swab specimens on two platforms: (1) the Cepheid GeneXpert Xpress System (Cepheid, Sunnyvale, CA), which tested for SARS-CoV-2, Influenza A and B (influenza A/B) viruses, and RSV, and (2) the BioFire FilmArray Respiratory Panel 2.1 (Salt Lake City, UT). We only included patients with positive test results within 3 days of admission for community-onset RSV infection. We then cross-referenced admission and discharge diagnosis codes to confirm that RSV-positive patients had a physician-diagnosed LRI using International Classification of Diseases, Tenth Revision (ICD-10) codes for bronchitis (J20.x), bronchiolitis (J21.x), and pneumonia (J09.X, J10, J12-18).^20,21^

To identify the comparison groups (IV and hMPV), we queried the Intermountain Healthcare’s Electronic Data Warehouse (EDW), a central repository of administrative and electronic medical data. Demographic, laboratory, and hospital data were abstracted. IV and hMPV-positive children with a physician diagnosed LRI were included as previously described for the RSV cohort. Abstracted data were vetted for eligibility by two study pediatricians (KA and PG).

To identify subsequent hospital readmissions among the 3 cohorts identified during the index RSV study period, the EDW was queried every 6 months to identify readmissions within a 1.5-year period from the discharge of the initial RSV, IV or hMPV LRI admission. Similarly, readmissions were evaluated by two pediatricians (KA and PG) to determine whether readmissions were respiratory-related or not. Children who were hospitalized for less than 24 h, and those with another viruses or bacteria detected by respiratory testing were excluded.

The study was reviewed and approved by the Intermountain Healthcare and University of Utah Institutional Review Boards (IRBs).

### Definition of Study Terms

All-cause readmission was defined as any readmission to hospital after discharge from the index hospital admission. Readmissions were classified as respiratory-related or non-respiratory-related based on chart review. A respiratory-related admission was defined as any clinical ailment related to the respiratory tract; a non-respiratory-related admission was defined as any clinical ailment unrelated to the respiratory tract.

### Statistical Analyses

We compared the characteristics of RSV, IV, and hMPV LRI cohorts, using two group *t-*test or its non-parametric countering part (Wilcoxon rank sum test) for continuous variables and Chi-squared test or Fisher’s exact test as appropriate for categorical variables. We calculated the crude incidence proportions for children with at least one all-cause and respiratory-related readmission within 30 days, 6 months, 1 year, and 1.5 years post-discharge, respectively. We assumed no patient attrition during the follow-up period. We used a Cox proportional hazards model with the inverse probability of treatment weighting using propensity scores to compare the hazards of having a readmission within 1.5 years post-discharge between the RSV, IV, and hMPV cohorts after controlling for confounders. Patients exited the study upon the occurrence of a study outcome of interest. Patients who did not experience the outcome event of interest were censored at the end of study periods (ie, 1.5 years of follow-up) if they did not die during their index admission. If patients died during their index admission, they were removed from the study. The list of confounders included age, gender, race, insurance status, presence of any chronic medical condition (CMC) and any respiratory or cardiovascular medical condition at readmission, as well as the requirement for intensive care unit (ICU) admission and the Length of Stay (LOS) during index hospitalization. The propensity score was calculated using the Covariate Balancing Propensity Score (CBPS) approach. The standardized mean differences (SMDs) were calculated and summarized using the love plot to show the change of covariate balance before and after CBPS adjustment. If the absolute SMDs for all covariates were lower than threshold value of 0.1, the covariate balances were achieved after adjustment. The adjusted hazard ratio and its 95% confidence interval were reported. All those analyses were performed using statistical software R.

## Results

### Patient Characteristics During Index Admission

**Figure 1.**
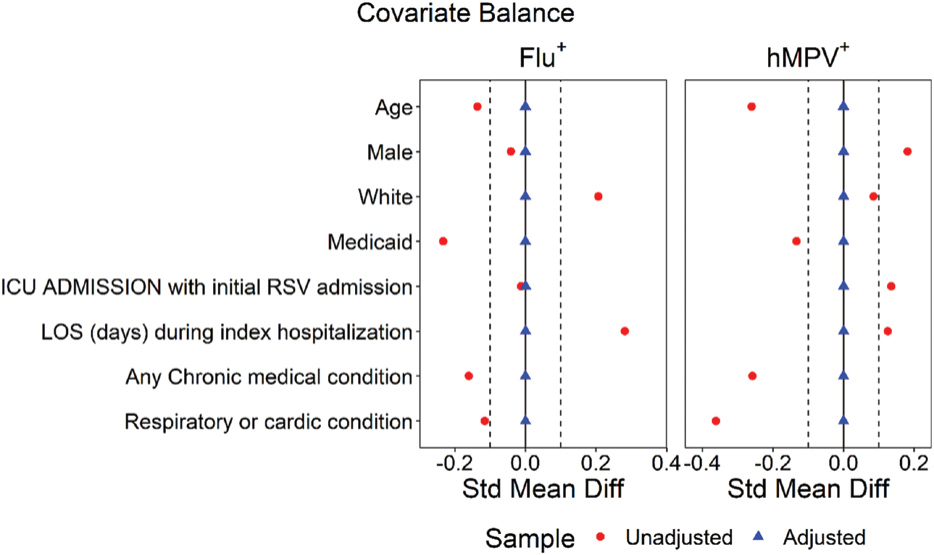
Covariate Balance Before and After Adjustment.

During the RSV-index season, 656, 43, and 68 hospitalized children with RSV, IV, and hMPV LRI, respectively, were identified. Demographics and characteristics of children with and without readmission after the respective index hospitalization by respiratory virus is summarized in **Table 1**. A significantly higher proportion of hospitalized children with RSV were younger than 6 months-of-age compared to IV (44% vs 23%; *P* = .01) and hMPV (44% vs 16%; *P* = .0001) patients. Compared to children with RSV, a higher proportion of children with hMPV had 2 or more CMC (22% vs 9%; *P* < .001), while children with IV had a shorter median LOS at their index admission (1.9 vs 1.3 days; *P* < .001). CBPS accounted for these differences in pairwise comparisons between children with RSV, IV, and hMPV (**Figure 1**).

**Table 1.** Demographics and Characteristics during Index Admission for Children Younger than 5 years, Hospitalized with Laboratory-confirmed RSV, IV, and hMPV Lower Respiratory Tract Infection, and Aeadmitted within 1.5 years Post-discharge after Index Admission.

	RSV			IV			hMPV		
	All^a^ (656)	No readmission (532)	Readmission^b^ (124)	All^a^ (43)	No readmission (30)	Readmission^b^ (13)	All^a^ (68)	No readmission (48)	Readmission^b^ (20)
Season									
10/2019 to 5/2020	214 (33%)	184 (35%)	30 (24%)^c^	33 (77%)^c^	24 (80%)	9 (69%)	32 (47%)^c^	22 (46%)	10 (50%)
6/2020 to 4/2021	1 (0.2%)	1 (0.2%)	–	–	–	-	1 (1.5%)	1 (2.1%)	–
5/2021 to 4/2022	441 (67%)	347 (65%)	94 (76%)	10 (23%)	6 (20%)	4 (31%)	35 (51%)	25 (52%)	10 (50%)
Age on index admission (months)									
<6	286 (44%)	234 (44%)	52 (42%)	10 (23%)^c^	8 (27%)	2 (15%)	11 (16%)^c^	5 (10%)	6 (30%)
6 to <12	91 (14%)	74 (14%)	17 (14%)	8 (19%)	5 (17%)	3 (23%)	14 (21%)	13 (27%)	1 (5.0%)
12 to <24	139 (21%)	109 (20%)	30 (24%)	14 (33%)	8 (27%)	6 (46%)	24 (35%)	16 (33%)	8 (40%)
24 to <60	140 (21%)	115 (22%)	25 (20%)	11 (26%)	9 (30%)	2 (15%)	19 (28%)	14 (29%)	5 (25%)
Mean (Std)	13.8 (14.1)	13.8 (14.2)	13.6 (13.5)	15.5 (10.9)	15.8 (11.7)	14.8 (9.1)	16.9 (10.0)^d^	17.3 (9.5)	16.1 (11.3)
Median (IQR)	8.5 (2.5, 21.2)	8.3 (2.4, 21.2)	8.8 (2.9, 19.5)	14.7 (7.1, 24.3)	15.0 (3.7, 27.2)	14.7 (7.7, 18.6)	16.2 (9.7, 25.6)	16.2 (10.4, 25.6)	15.9 (4.9, 24.3)
Male (%)	368 (56%)	288 (54%)	80 (65%)^e^	25 (58%)	19 (63%)	6 (46%)	32 (47%)	22 (46%)	10 (50%)
Race (%)									
White	432 (66%)	350 (66%)	82 (66%)	24 (56%)	17 (57%)	7 (54%)	42 (62%)^c^	28 (58%)	14 (70%)
PI/NH	49 (7.5%)	37 (7.0%)	12 (9.7%)	5 (12%)	2 (6.7%)	3 (23%)	9 (13%)	6 (13%)	3 (15%)
Asian	16 (2.4%)	13 (2.4%)	3 (2.4%)	4 (9.3%)	4 (13%)	–	4 (5.9%)	3 (6.3%)	1 (5.0%)
Black	17 (2.6%)	13 (2.4%)	4 (3.2%)	1 (2.3%)	1 (3.3%)	–	5 (7.4%)	3 (6.3%)	2 (10%)
Other	142 (22%)	119 (22%)	23 (19%)	9 (21%)	6 (20%)	3 (23%)	8 (12%)	8 (17%)	–
Insurance (%)									
Commercial	243 (37%)	203 (38%)	40 (32%)^c^	12 (28%)	11 (37%)	1 (7.7%)	27 (40%)	21 (44%)	6 (30%)
IH insurance	121 (18%)	104 (20%)	17 (14%)	6 (14%)	3 (10%)	3 (23%)	6 (8.8%)	3 (6.3%)	3 (15%)
Medicaid	275 (42%)	209 (39%)	66 (53%)	23 (53%)	14 (47%)	9 (69%)	33 (49%)	22 (46%)	11 (55%)
Self-pay	17 (2.6%)	16 (3.0%)	1 (0.8%)	2 (4.7%)	2 (6.7%)	–	2 (2.9%)	2 (4.2%)	–
Any CMC (%)	138 (21%)	102 (19%)	36 (29%)^e^	12 (28%)	5 (17%)	7 (54%)^c^	22 (32%)^e^	14 (29%)	8 (40%)
>/=2 CMC (%)	60 (9.1%)	41 (7.7%)	19 (15%)^e^	6 (14%)	2 (6.7%)	4 (31%)	15 (22%)^e^	7 (15%)	8 (40%)^c^
Neurological (%)	18 (2.7%)	11 (2.1%)	7 (5.6%)	3 (7.0%)	1 (3.3%)	2 (15%)	3 (4.4%)	–	3 (15%)^c^
Cardiovascular (%)	63 (9.6%)	42 (7.9%)	21 (17%)^e^	6 (14%)	2 (6.7%)	4 (31%)	15 (22%)^e^	6 (13%)	9 (45%)^c^
Respiratory (%)	23 (3.5%)	14 (2.6%)	9 (7.3%)^c^	2 (4.7%)	0 (0%)	2 (15%)	6 (8.8%)^c^	4 (8.3%)	2 (10%)
Renal (%)	17 (2.6%)	12 (2.3%)	5 (4.0%)	3 (7.0%)	2 (6.7%)	1 (7.7%)	4 (5.9%)	–	4 (20%)^c^
Gastrointestinal (%)	32 (4.9%)	19 (3.6%)	13 (10%)^e^	5 (12%)	1 (3.3%)	4 (31%)^c^	8 (12%)^c^	2 (4.2%)	6 (30%)^c^
Hematologic (%)	6 (0.9%)	3 (0.6%)	3 (2.4%)	2 (4.7%)	–	2 (15%)	3 (4.4%)^c^	2 (4.2%)	1 (5.0%)
Genetic/Metabolic (%)	56 (8.5%)	38 (7.1%)	18 (15%)^*c^	7 (16%)	1 (3.3%)	6 (46%)^c^	16 (24%)^*c^	10 (21%)	6 (30%)
Malignancy (%)	3 (0.5%)	2 (0.4%)	1 (0.8%)	1 (2.3%)	–	1 (7.7%)	1 (1.5%)	1 (2.1%)	–
h/o prematurity (<37 weeks) (%)	74 (11%)	47 (8.8%)	27 (22%)^e^	2 (4.7%)	1 (3.3%)	1 (7.7%)	11 (16%)^c^	4 (8.3%)	7 (35%)
Transplant (%)	–	–	–	2 (4.7%)^c^	–	2 (15%)	1 (1.5%)	–	1 (5.0%)
LOS during index admission									
Mean (Std)	2.8 (3.1)	2.8 (3.0)	3.1 (3.8)	1.9 (3.2)	1.3 (1.4)	3.5 (5.2)	2.4 (3.0)	2.1 (2.3)	3.4 (4.1)
Median (IQR)	1.9 (1.1, 3.1)	1.9 (1.2, 3.0)	1.8 (1.1, 3.2)	1.3 (0.6, 1.8)^f^	1.1 (0.5, 1.4)	1.3 (1.0, 2.8)	1.5 (1.0, 2.6)^g^	1.3 (0.9, 2.4)	2.3 (1.2, 3.6)
ICU admission (%)	119 (18%)	96 (18%)	23 (19%)	8 (19%)	5 (17%)	3 (23%)	9 (13%)	5 (10%)	4 (20%)

Abbreviations: IH insurance, Intermountain Health insurance; CMC, chronic medical condition; Std, standard deviation; IQR, interquartile range; h/o, history of; ICU, intensive care unit; PI/NH, Pacific Islander/Native Hawaiian, LOS, length of stay.

^a^Comparison between overall RSV vs overall FLU, overall RSV vs overall HMPV.

^b^Comparison within RSV, FLU, and HMPV, comparing the distribution between patients with and without readmission.

^c^Fisher’s exact test.

^d^ *t*-test.

^e^Chi-squared test.

^f^Exact Wilcoxon rank sum test.

^g^Wilcoxon rank sum test.

^*^ *P* < .05.

### Incidence Proportion and Hazard Ratio of Hospital Readmission

Over the 1.5-year follow-up study period, 124, 13, and 20 children with RSV, IV, and hMPV respectively had a readmission, with 42, 5, and 3 children respectively having ≥2 readmissions. The median age at readmission increased to 15.2 (interquartile range, IQR: 5.8-25.7), 19.6 (IQR: 11.3-28.1), and 23.9 (IQR: 12.3-31), months, respectively (data not shown).

During their index RSV hospitalization, the children who were later readmitted were predominantly male, had non-commercial insurance, and had at least one CMC compared to those who were not readmitted. Similarly, readmitted children after their IV or hMPV hospitalization, were more likely to have at least one or multiple CMCs during their index admission, respectively, compared to those who were not readmitted. (**Table 1**). The 30-day incidence proportion of all-cause readmission for children with RSV, IV, and hMPV LRI hospitalization was 7.0%, 4.7%, and 8.8%, respectively, which increased progressively over time to 18.9%, 30.2%, and 29.4% within 1.5 years post-discharge (**Table 2**). All-cause readmission proportions were higher for IV (*P* < .05) within 1 year and for hMPV (*P* < .05) within 1.5 years post-discharge when compared to RSV. Median duration to the first all-cause RSV readmission (88 days) was shorter compared to IV (187 days) and hMPV (196 days) (**Table 3**). However, after adjusting for CBPS, there were no significant differences associated with the risk of all-cause readmission in the RSV cohort compared to the IV (HR 0.67, 95% CI, 0.35-1.29) and hMPV (HR 0.63, 95% CI, 0.38-1.06) cohorts.

**Table 2.** Crude Cumulative Incidence Proportion of the First All-Cause and Respiratory-related Readmission among Children Younger than 5 Years of Age Previously Hospitalized at PCH and RH with RSV-, IV- and Hmpv LRI and Followed for 1.5 years Post-discharge.

Readmission	RSV (95% CI)	IV (95% CI)	hMPV (95% CI)
**All cause (*n* within 1.5 years)**	**124**	**13**	**20**
**30-day**	7.0% (5.3%-9.3%)	4.7% (1.2%-18%)	8.8% (4.1%-18.9%)
**6 months**	12.3% (10.1%-15.1%)	11.6% (5.1%-26.5%)	14.7% (8.3%-26.1%)
**1 year**	15.7% (13.2%- 18.7%)	27.9% (17.3%-45.1%)^*^	22.1% (14.1%-34.5%)
**1.5 year**	18.9% (16.1%-22.1%)	30.2% (19.2%-47.6%)	29.4% (20.4%-42.5%)^*^
**Respiratory-related (*n* within 1.5 years)**	**110**	**3**	**4**
**30-day**	6.3% (4.6%-8.4%)	–	–
**6 months**	11.4% (9.2%-14.1%)	–	–
**1 year**	14.0% (11.6%-17.0%)	7% (2.3%,20.8%)	4.4% (1.5%,13.3%)^*^
**1.5 year**	16.8% (14.1%-19.9%)	7% (2.3%,20.8%)	5.9% (2.3%,15.2%)^*^

^*^ *P* < .05 for RSV vs. IV or RSV vs. hMPV.

**Table 3.** Median Duration (days) to the First All-Cause and Respiratory-related Readmission and Adjusted Hazard Ratio Among Children Younger than 5 Years of Age Previously Hospitalized at PCH and RH with RSV-, IV- and hMPV LRI.

Readmission	Median duration (days) to first readmission (IQR)			Hazard ratios (95% CI)	
	RSV	IV	hMPV	RSV vs. IV (ref)	RSV vs. hMPV (ref)
**All-cause**	88.1 (6.1, 283.4)	187.0 (51.5, 255.6)	196.4 (23.0, 311.8)	0.67 (0.35, 1.29)	0.63 (0.38, 1.06)
**Respiratory-related**	74.4 (4.9, 272.6)	255.6 (236.3, 255.7)	264.6^*^ (255.7, 334.5)	3.62 (1.13, 11.64)	3.56 (1.14, 11.06)

^*^ *P* < .05 for RSV vs. hMPV.

Readmissions for 110 (89%), 3 (23%), and 4 (20%) children with RSV, IV, and hMPV, respectively, were assessed to be respiratory related. Respiratory-related readmission proportions were significantly higher among RSV children compared to IV (89% vs 23%; *P* < .0001) and hMPV (89% vs 20%; *P* < .0001), respectively. The 30-day incidence respiratory-related readmission proportion of children with RSV was 6.3% (95% CI, 4.6-8.4) and increased progressively over the study period to 16.8% (95% CI, 14.1-19.9) within 1.5 years post-discharge. While 69% of RSV respiratory-related readmissions occurred within the first 6 months after discharge, there were no respiratory-related readmissions among children with IV and hMPV during that period. Respiratory-related readmissions occurred later in these children and were overall lower for IV (7% vs 16.8%; *P* = .06) and hMPV (5.9% vs 16.8%; *P* = .03) when compared to RSV within 1.5 years post-discharge (**Table 2**). The median duration to the first respiratory-related readmission was shorter for RSV (74 days), compared to IV (256 days) and hMPV (265 days, *P* < .05). After adjusting for CBPS, an initial admission for RSV LRI was associated with a >3-fold increased risk of a respiratory-related readmission compared to IV (HR 3.62, 95% CI, 1.13-11.64) or hMPV (HR 3.56, 95% CI, 1.14-11.06) (**Table 3**). Among patients with a respiratory-related readmission after RSV, the average hospital stay was 2.2 days, and 16% of these patients required intensive care.

The association between age at initial admission with RSV and subsequent first respiratory-related readmission was pronounced in children ≤12 months (**Table S1**). A summary of discharge diagnoses of respiratory-related readmissions of children with RSV, IV, and hMPV are summarized in **Table S2**, bronchiolitis or asthma accounting for a majority (91%) of RSV respiratory-related readmissions.

## Discussion

Respiratory viral infections in early childhood are common and have been implicated in short- and long-term health consequences that result in hospital readmissions. Our study is one of the few to evaluate and compare hospital readmissions following LRI hospitalization by RSV, IV, and hMPV. In our study, all-cause readmissions were common after RSV, IV, and hMPV LRI hospitalization, with a 30-day all-cause readmission proportions, ranging between 5% and 9%, and increasing to between 19% and 30% 1.5-years post-discharge. Most readmissions were respiratory-related, and higher among children with RSV compared to IV and hMPV. Respiratory-related readmissions occurred much earlier among children with RSV, with the 30-day RSV respiratory-related readmission proportion was 6.3%, and no readmissions among IV and hMPV children in this timeframe. Respiratory-related readmissions among children with RSV progressively increased to 16.8%, 1.5-years post-discharge, which was higher than that among children with IV (7%) and hMPV (5.9%) 1.5-years post-discharge. Our data show that RSV LRI hospitalization was associated with an increased hazard of a respiratory-related readmission of 3.62 (95% CI, 1.13-11.64) and 3.56 (95% CI, 1.14-11.06) compared to IV and hMPV LRI, respectively.

In our study, the 30-day all-cause readmission proportions for children hospitalized with RSV, IV, and hMPV are comparable to earlier studies reporting 30-day readmission rates of 5.2%-7.7% among children hospitalized with LRI, pneumonia, and bronchiolitis.^22-24^ While few studies have evaluated long-term outcomes of children hospitalized with LRI, our all-cause readmission proportions for RSV after 1 and 1.5 years post-discharge are similar in magnitude to readmissions proportions reported by Pelletier et al. who, using the Pediatric Health Information Systems (PHIS) database reported a 1-year readmission proportion of 17.8% among children <2 years hospitalized with bronchiolitis. In our study, the median time to all-cause RSV readmission was 88 days, also in keeping with observations made by Pelletier et al. of readmissions that increased and plateaued at four months after hospital discharge.^23^ The similarity in both studies can be attributed to the fact that ~80% of bronchiolitis hospitalizations are caused by RSV.**^25^** Despite other studies using different study end points, our 30-day all-cause IV readmission proportion of 4.7% is comparable to a study by Brogan et al., who reported a 30-day readmission proportion of 4.7% and 10.3% among children hospitalized with uncomplicated and complicated H1N1 influenza infection.**^26^** In contrast, among children with hMPV, our all-cause readmission proportion of 30% after 1.5 years, is higher than the 23% readmission proportion for acute respiratory illness reported among children <2 years and followed for 2 years.^27^

Short- and long-term consequences of recurrent wheezing and asthma have been reported following respiratory viral LRI in children.^13-16^ Proposed mechanisms for recurrent wheezing and asthma after RSV and RV infection include chronic epithelial and airway reactivity changes that alter lung function, immunomodulatory changes, and or genetic factors that impact patterns of immune response.^14,28,29^ In our study, most of respiratory-related readmissions were associated with bronchiolitis or asthma, similar to Nakamura et al. who reported LRI (48.2%), asthma (10%), and respiratory failure (3.3%) as primary diagnoses associated with the 30-day readmission after LRI hospitalization.^22^

Our study demonstrated a >3-fold increased hazard of a respiratory-related readmission for children with RSV compared to children with IV and hMPV during early childhood. We adjusted disease severity^30,31^ at index hospitalization and age^32^ that were suggested as possible confounders of readmission. Younger age, as observed in our exploratory analysis, is potentially linked to an immature immune response, smaller airways, and increased inflammatory cytokines in the respiratory tract caused by RSV compared to IV and hMPV. Furthermore, there are other poorly understood differences in the pathogenesis of these infections.^33^ Animal studies may also provide some clues to the observed differences in our study. Among mice infected with RSV and hMPV, RSV replicated to higher titers than hMPV in the lung and in the upper respiratory tract, and virus elimination from the lungs was more rapid in hMPV-infected mice. Also, while there was similar recruitment of T lymphocytes (predominance of IFN-γ-producing CD8 + T cells), there were differences in the innate immune response, with hMPV infection resulting in more neutrophils in the airways and more activated NK cells than in RSV-infected mice, which correlated with higher levels of IL-6, TNF-α and MCP-1, and possible, earlier clearance of hMPV from the respiratory tract compared to RSV.^34^

In this study, a respiratory-related readmission following RSV hospitalization resulted in a hospitalization with a mean length of 2.2 days, and 16% requiring intensive care, demonstrating the healthcare burden. As a result of this hospital burden, admissions and readmissions for common pediatric illnesses, eg, bronchiolitis, have been a target for the Pediatric Quality Measures Program of the Medicare & Medicaid Services, and the Agency for Healthcare Research, with a number of care process models developed to reduce readmission of children previously hospitalized with LRI and bronchiolitis.^35,36^ Our study findings emphasize the importance of addressing the burden of RSV-related hospitalizations through effective prevention measures. The additional economic burden associated with the subsequent readmissions should be taken into consideration by policymakers when assessing the cost-effectiveness of new RSV prevention strategies.

Our study has several strengths. First, we conducted active surveillance with routine respiratory viral testing for all hospitalized children with respiratory symptoms. Additionally, our study periods overlapped with the COVID pandemic that started in early March 2020 in Utah. This increased testing propensity for respiratory viral infections, reducing the risk of exposure misclassification (ie, RSV cases that went untested and undiagnosed). Similarly, except for the first season (October 31, 2019 to May 1, 2020), we ascertained RSV, IV, and hMPV cases year-round, mitigating the impact of COVID-interrupted RSV seasonality. Second, our 1.5-year follow-up period covered subsequent winter seasons, allowing ascertainment of short-term and long-term readmissions. Finally, we balanced a comprehensive set of underlying risk conditions across comparison groups by using CBPS.

There were some limitations in our study. Firstly, we were not able to evaluate the attrition rate during the 1.5-year follow-up period. However, Primary Children’s and Riverton Hospital serve as community and tertiary hospitals for Salt Lake City, Utah, and the surrounding Intermountain Health region. We believe that the potential attrition rate would be low and non-differential across comparison groups. Secondly, we used a standardized time window to identify index admissions, which was anchored to RSV seasonality. This was necessary to assume the same parental health-seeking behavior (eg, willingness to visit, be evaluated and be admitted to hospital), which could vary depending on the phases of COVID pandemic and causing potential outcome misclassification. However, since the seasons for hMPV, RSV, and IV do not precisely overlap, the generalizability of index admissions included for the IV or hMPV groups could be affected. Thirdly, while previous studies have evaluated readmissions following RV LRTI infection, the study could not compare and contrast readmissions following RSV with RV or clarify the relative risk of readmission compared to other common respiratory viruses. Fourthly, we followed patients for 1.5 years after index admission, and it is possible longer follow-up may have impacted our study results. Finally, the generalizability of our study results may be limited, and it is important to interpret the findings within the context of viral seasonality, physician’s testing/diagnostic practices, and patient health-seeking behavior.

## Conclusion

Respiratory-related readmission proportion was higher and progressive over time among children <5 years with an index RSV admission compared to IV and hMPV. This underscores the critical need for prevention of RSV infection in infants and young children with maternal RSV vaccines or RSV monoclonal antibodies.

## Supplementary Material

piaf036_suppl_Supplementary_Tables_S1-S2
