# Supplementary material for: Readmission Following Respiratory Syncytial Virus Hospitalization among Children <5 Years of Age
Source: J Pediatric Infect Dis Soc. 2025 Apr 29;14(5):piaf036. doi: 10.1093/jpids/piaf036 (PMC12123188; doi:10.1093/jpids/piaf036)
Supplement: piaf036_suppl_Supplementary_Tables_S1-S2 [file piaf036_suppl_supplementary_tables_s1-s2.docx]

**Supplemental table 1. Age-stratified hazard ratio for respiratory-related readmission**

|  | **Hazard ratios (95% CI)** | |
| --- | --- | --- |
|  | **RSV vs. IV (ref)** | **RSV vs. hMPV (ref)** |
| **≤ 12 months** | 4.5 (0.61, 33.35) | - |
| **> 12 months** | 2.24 (0.44, 11.34) | 2.54 (0.88, 7.32) |

No respiratory-related readmission occurred following the index hMPV admission in infants ≤12 months-of-age.

**Supplemental table 2. Clinical syndromes associated with respiratory-related admissions among children 1.5 years after index hospitalization from RSV, IV and hMPV.**

|  | **RSV** | **IV** | **hMPV** |
| --- | --- | --- | --- |
| **Bronchiolitis with a respiratory virus*** | 77 | 1 | 2 |
| **Bronchiolitis without a respiratory virus** | 14 | 1 | - |
| **Asthma with a respiratory virus*** | 3 | - | - |
| **Asthma without a respiratory virus** | 6 | - | - |
| **Pneumonia with or without a respiratory virus*** | 10 | 1 | 2 |
| **Total** | **110** | **3** | **4** |

*Respiratory viruses include, RSV, IV (A and B), hMPV, adenovirus, parainfluenza (1-4), Coronavirus (HKU1, OC43, NL63, SARS-CoV-2), rhinovirus/enterovirus
